# Critical Role of the Rb Family in Myoblast Survival and Fusion

**DOI:** 10.1371/journal.pone.0017682

**Published:** 2011-03-10

**Authors:** Giovanni Ciavarra, Andrew T. Ho, David Cobrinik, Eldad Zacksenhaus

**Affiliations:** 1 Department of Laboratory Medicine and Pathobiology, University of Toronto, Toronto, Ontario, Canada; 2 Department of Pediatrics, Memorial Sloan-Kettering Cancer Center, New York, New York, United States of America; 3 Division of Cell and Molecular Biology, Toronto General Research Institute - University Health Network, Toronto, Ontario, Canada; University of Maastricht (UM), Netherlands

## Abstract

The tumor suppressor Rb is thought to control cell proliferation, survival and differentiation. We recently showed that differentiating Rb-deficient mouse myoblasts can fuse to form short myotubes that quickly collapse through a mechanism involving autophagy, and that autophagy inhibitors or hypoxia could rescue the defect leading to long, twitching myotubes. Here we determined the contribution of pRb relatives, p107 and p130, to this process. We show that chronic or acute inactivation of Rb plus p107 or p130 increased myoblast cell death and reduced myotube formation relative to Rb loss alone. Treatment with autophagy antagonists or hypoxia extended survival of double-knockout myotubes, which appeared indistinguishable from control fibers. In contrast, triple mutations in Rb, p107 and p130, led to substantial increase in myoblast death and to elongated bi-nuclear myocytes, which seem to derive from nuclear duplication, as opposed to cell fusion. Under hypoxia, some rare, abnormally thin triple knockout myotubes survived and twitched. Thus, mutation of p107 or p130 reduces survival of Rb-deficient myoblasts during differentiation but does not preclude myoblast fusion or necessitate myotube degeneration, whereas combined inactivation of the entire Rb family produces a distinct phenotype, with drastically impaired myoblast fusion and survival.

## Introduction

The retinoblastoma tumor suppressor (Rb) plays crucial roles in development and homeostasis, and is commonly inactivated in human malignancies [1], [2], [3]. Rb is a member of a family of proteins including p107 and p130 that exhibit similar or opposing functions in different tissues [4]. The Rb family is thought to control cell proliferation and survival by binding E2F family of transcription factors and repressing transcription by recruiting chromatin-modifying factors such as HDAC1 [5]. The Rb family may also regulate differentiation by controlling expression of differentiation factors such as PPARγ and PGC-1α and by sequestering inhibitors of differentiation including ID2, HDAC1, EID1 and RBP2 [6], [7], [8], [9], [10], [11]. Rb in particular was shown to potentiate the activity of lineage-specific transcription factors such as the myogenic factors MyoD and myogenin during skeletal myogenesis [12], [13], [14], [15], [16], [17]. These myogenic proteins bind promoters of muscle-specific genes like muscle creatine kinase (MCK) to activate the muscle differentiation program [18], [19], [20], [21]. Indeed, ectopic expression of MyoD in Rb-deficient fibroblasts fails to induce myogenesis [22], [23].

In keeping with Rb's ability to potentiate myogenic conversion in fibroblasts, pRb is required for proper skeletal myogenesis *in vivo*. Rb-null embryos die at embryonic day (E) 13.5–14.5, exhibiting ectopic proliferation, massive apoptosis and incomplete differentiation in a number of tissues where Rb is normally highly expressed [24], [25], [26], [27], [28], [29], [30]. The early embryonic death precluded studies of terminal skeletal myogenesis, which occurs after embryonic day (E) 14.5. An Rb mini-gene (mgRb), expressed exclusively in the placenta and the nervous system, but not in skeletal muscles, was used to extend the life-span of Rb^−/−^ embryos to birth [31], [32](Z. Jiang and EZ, unpublished). In mgRb:Rb^−/−^ embryos, myotubes are initially formed at E14.5–15.5, but thereafter degenerate in a process accompanied by massive myoblast apoptosis, endoreduplication within myotubes and normal expression of early myogenic differentiation markers (MHC, cardiac actin) but not late markers (MCK, MRF4). Similar muscle defects were subsequently reported after Rb-null embryos were partially rescued by other means [8], [33], [34]. Moreover, a muscle-specific ablation of a floxed Rb allele (Rb^f^) via Myf5-Cre demonstrated impaired muscle differentiation both *in vitro* and *in vivo* [35]. Finally, analysis of Rb mutant myoblasts (*in vitro*) revealed a similar pattern observed in Rb mutant fetuses (*in vivo*); that is, myoblasts initially fuse to form short myotubes but quickly degenerate and never twitch [36].

Although these observations implicate pRb in terminal myogenesis, a direct assignment of differentiation function to pRb was hampered by the fact that terminal differentiation is intimately coupled to myoblast survival. To overcome this obstacle, the ability of Rb mutant myoblasts to differentiate has been assessed in the presence of survival factors. Remarkably, expression of Bcl-2 rescued the Rb defect leading to long myotubes that twitched for weeks in culture [36]. Furthermore, differentiating Rb-deficient myotubes exhibited perinuclear mitochondrial aggregation and autophagy, not apoptosis, and inhibition of autophagy or exposure to hypoxia suppressed myotube degeneration [36]. Although differentiating Rb mutant myotubes initially failed to exit the cell cycle, the rescued myotubes eventually became stably post-mitotic despite absence of Rb. Together these results suggest that Rb is required to coordinate cell cycle exit with survival during the onset of differentiation, but not for actively stimulating the differentiation program.

An important unresolved question is whether p107 and p130 compensate or exacerbate the differentiation defect of Rb-deficient myoblasts. In particular, we wish to know whether the initial cell fusion and myotube formation that occurs in the absence of pRb is p107 and/or p130-dependent. There are several notable differences among Rb family members, including their affinity to E2F members and other factors [5], [37], effects on cell cycle exit and senescence [38], , and roles during embryogenesis and cancer [24], [40], [41], [42], [43], [44], [45], [46], [47]. During myogenesis, over-expression of p107 suppressed ectopic cell proliferation in Rb^−/−^ myotubes [16], whereas over-expression of p130, but not pRb, inhibited myogenic differentiation in Rb proficient mouse C2 myoblasts [48]. These findings raised the possibility that p107 might act in a manner redundant to Rb, whereas p130 might promote defects resulting from Rb deficiency during myogenic differentiation.

To address the function of the Rb protein family during terminal differentiation, we analyzed double Rb^−/−^:p107^−/−^ and Rb^−/−^:p130^−/−^ as well as triple Rb^−/−^:p107^−/−^:p130^−/−^ mutant myoblasts following chronic or acute inactivation of Rb. We report that loss of either p107 or p130 enhanced the survival defect of Rb-deficient myoblasts, implying that both of these Rb-related proteins partially compensate for Rb loss. Nevertheless, myoblasts lacking Rb plus either p107 or p130 could differentiate and twitch, as long as autophagic cell death is suppressed or metabolism is shifted to glycolysis under hypoxia. In contrast, myoblasts lacking all three Rb family members do not efficiently fuse or survive, indicating that expression of at least one Rb family protein is essential for these processes.

## Results

### Combined mutation in p130 does not counteract Rb myogenic defects in mgRb:Rb^−/−^:p130^−/−^ double mutant fetuses

As noted, over-expression of p107 suppresses ectopic cell proliferation in Rb^−/−^ myotubes [16], whereas over-expression of p130 inhibits myogenic differentiation in Rb proficient mouse C2 myoblasts [48], suggesting that combined loss of Rb plus p107 or Rb plus p130 might worsen or ameliorate the Rb myogenic defect, respectively. To test the effects of p107 and p130 on differentiation of Rb-deficient myoblasts, we generated and intercrossed mgRb:Rb^+/−^:p107^+/−^ and mgRb:Rb^+/−^:p130^+/−^ double heterozygote mice. Consistent with previous studies on early death of Rb/p107 double mutant embryos [42], [49], we were unable recover live E14.5–E16.5 mgRb:Rb^−/−^:p107^−/−^ double mutant embryos after breeding mgRb:Rb^+/−^:p107^+/−^ mice (not shown). In contrast, E14.5–E16.5 mgRb:Rb^−/−^:p130^−/−^ fetuses were identified following mgRb:Rb^+/−^:p130^+/−^ interbreeding at the expected Mendelian frequency (6/53 = 11.32%; expected 12.5%; **Table 1**). At E17.5, the frequency of mgRb:Rb^−/−^:p130^−/−^ double knockout (KO) embryos dropped to 1/59 (1.69%), whereas viable Rb^−/−^ single knockouts were present at 9/59 (24%), approximating the expected 25% frequency.

**Table 1:** Frequency of *mgRb:Rb^−/−^*:*p130^−/−^* embryos recovered.

Gestation	*mgRb:Rb^−/−^* *	*mgRb:Rb^−/−^:p130^−/−^* *	*mgRb:Rb^−/−^:p130^−/−^* **
E14.5	4/54 (7.41%)	6/54 (11.11%)	Not determined
E15.5	8/41 (19.51%)	6/41 (14.63%)	6/38 (15.79%)
E16.5	11/53 (20.75%)	6/53 (11.32%)	2/20 (10.0%)
E17.5	9/59 (24.09%)	1/59 (1.69%)	4/45 (8.89%)

*Mutant embryos obtained from *mgRb:Rb^+/−^:p130^+/−^* × *mgRb:Rb^+/−^:p130^−/−^* intercrosses. Total of 5 *in utero* deaths at E17.5 were not included. A frequency of 12.5% is expected in each indicated group.

**Mutant embryos obtained from *mgRb:Rb^+/−^:p130^−/−^* × *mgRb:Rb^+/−^:p130^−/−^* intercrosses. Total of 4 *in utero* deaths at E17.5 were not included. A frequency of 25% is expected in *mgRb:Rb^−/−^:p130^−/−^* group.

**Figure 1:**
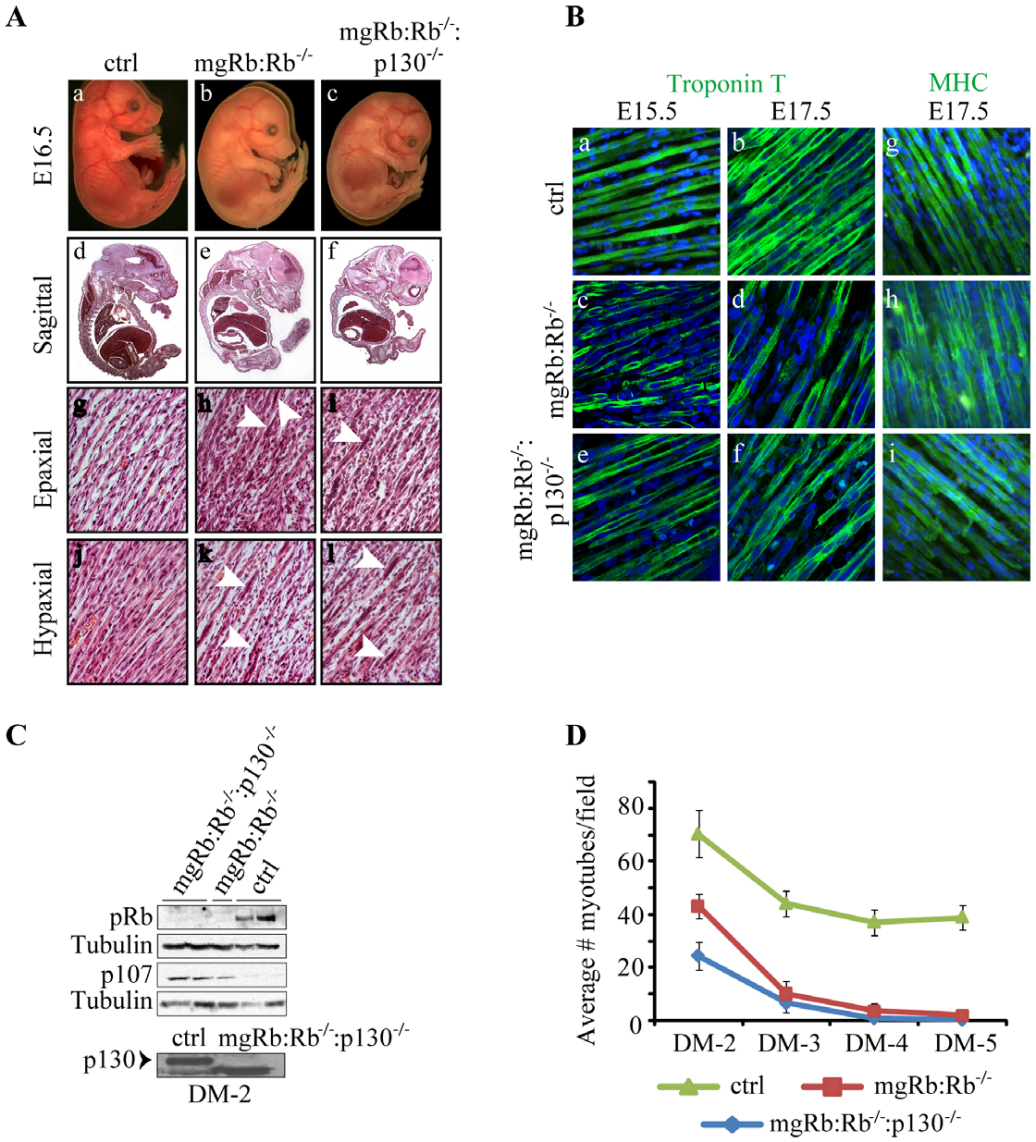
Analysis of myogenesis mgRb:Rb^−/−^:p130^−/−^ double mutant embryos. (A) Appearance of ctrl, mgRb:Rb^−/−^ and mgRb:Rb^−/−^:p130^−/−^ embryos at E16.5 (a–c). Hematoxylin & eosin (H&E) histology staining reveals the cellular morphology at mid-sagittal section (d–f), epaxial muscles (g–i) and hypaxial muscles (j–l). Arrowheads point to enlarged nuclei within myofibers. (B) Confocal images showing fast troponin T (green) expression in skeletal muscle sections of control (ctrl) (a–b) mgRb:Rb^−/−^ (c–d) and mgRb:Rb^−/−^:p130^−/−^ (e–f) embryos. MHC (green) expression in skeletal muscle sections of ctrl (g) mgRb:Rb^−/−^ (h) and mgRb:Rb^−/−^:p130^−/−^ (i) embryos. DAPI was used to counter-stain nuclei (blue). (C) Top, western blot analysis for pRb and p107 in DM-2 ctrl, mgRb:Rb^−/−^ and mgRb:Rb^−/−^:p130^−/−^ myoblast cultures. Tubulin was used as loading control. Bottom, western blot analysis of p130 in DM-2 ctrl and mgRb:Rb^−/−^:p130^−/−^ cultures. Arrow indicates location of p130. Lower band represents a splice variant or cross-reactive protein. (D) Average number of myotubes counted on indicated days post-differentiation. Each time point represents an average ± s.d. of 6 fields at 200X (n = 4).

Compared to E16.5 mgRb:Rb^−/−^ single KO embryos, mgRb:Rb^−/−^:p130^−/−^ double knockout (DKO) fetuses displayed a more pronounced hunchback, suggesting reduced muscle toning (**Fig. 1A**, top panels). However, histological sections through epaxial and hypaxial skeletal muscles of mgRb:Rb^−/−^:p130^−/−^ fetuses revealed similar defects as in mgRb:Rb^−/−^ littermates, including reduced density and shortened myofibers, and enlarged nuclei within myotubes compared to control (**Fig. 1A**, arrowheads). Expression of myosin heavy chain (MHC), an early marker of differentiation, was similar in wild type and mutant embryos whereas expression of troponin T, a late marker of differentiation, was similarly reduced in single and double mutants relative to control (**Fig. 1B**). Terminal deoxynucleotidyl transferase biotin-dUTP nick end labeling (TUNEL, [50]) analysis revealed no obvious differences in apoptosis: 22.2±9.3% and 20.1±4.7% for single and DKO, respectively (not shown). Thus, despite the enhanced hunchback mgRb:Rb^−/−^:p130^−/−^ DKO embryos, we did not detect obvious changes in myoblast differentiation *in vivo*.

### Combined mutations in Rb and p130 accelerated myotube degeneration *in vitro*

To test for a cell-autonomous effect of combined mutations in Rb and p130, we derived primary myoblasts from E16.5 mgRb:Rb^−/−^:p130^−/−^ limb muscles. Absence of pRb and p130 was verified in mgRb:Rb^−/−^:p130^−/−^ cultures 2 days post-differentiation (DM-2) (**Fig. 1C**). We note that the p130^−/−^ lane lacked full-length p130, but contained a lower band, which could represent a cross reactive protein or a truncated/spliced p130 variant. Expression of p107, which was undetected in DM-2 control myotube cultures, was elevated in Rb KO myotubes and further increased in Rb/p130 DKO cultures (**Fig. 1C**). This observation is consistent with the presence of E2F binding sites in the p107 promoter and its regulation by pRb [51], [52], and may partly compensate for the combined loss of pRb and p130 in these DKO cells.

Under differentiation conditions, wild-type myoblasts fused to form long multinucleated myotubes that twitched for weeks in culture (**Fig. 1D**). In contrast, both mgRb:Rb^−/−^ and mgRb:Rb^−/−^:p130^−/−^ myoblasts initially fused to form short myotubes containing 3–6 nuclei, but underwent rapid degeneration beginning 2–3 days post-differentiation; by day 5–6 virtually all myotubes had degenerated. Reproducibly, the mgRb:Rb^−/−^:p130^−/−^ DKO myoblasts exhibited reduced myotube formation relative to mgRb:Rb^−/−^ cultures, but similar degeneration kinetics (**Fig. 1D**).

**Figure 2:**
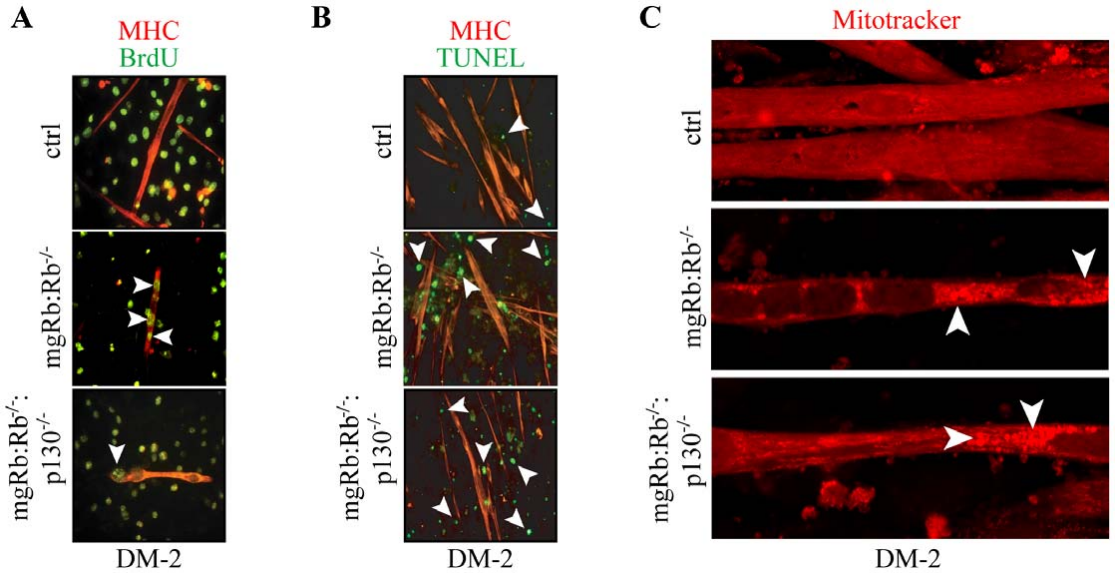
Differentiation of mgRb:Rb^−/−^:p130^−/−^ DKO myoblasts. (A) Confocal microscopy analysis for BrdU incorporation in ctrl, mgRb:Rb^−/−^ and mgRb:Rb^−/−^:p130^−/−^ myotubes at DM-2. Myoblasts were differentiated for 1 day, then exposed to 20 µM BrdU for an additional 16 hr in the presence of growth medium (GM) and immuno-stained for MHC (red) and BrdU (green). Arrowheads label BrdU positive nuclei within myotubes. (B) MHC (red) and TUNEL (green) staining at DM-2. Arrowheads indicate TUNEL positive nuclei, which are invariably located outside myotubes. (C) Mitotracker® (red) staining at DM-2. Arrowheads point to large Mitotracker®-positive perinuclear aggregates.

Unlike wild-type myotubes, which are stably post-mitotic (**Fig. 2A**, top), nuclei in Rb-deficient myotubes incorporate BrdU when stimulated with mitogens, indicating a failure in establishing a permanent cell cycle exit (**Fig. 2A**, middle) [16], [23], [36]. To test the cell cycle status of DKO myotubes, cultures were differentiated for 1 day and then re-stimulated with growth medium in the presence of BrdU. Like Rb^−/−^, the mgRb:Rb^−/−^:p130^−/−^ myotubes incorporated BrdU (**Fig. 2A**, bottom). We next tested whether mgRb:Rb^−/−^:p130^−/−^ myotube degeneration was associated with apoptosis. TUNEL-positive nuclei were detected in unfused myoblasts, but not within myotubes, and were more abundant in Rb and Rb/p130 DKO cultures than in control (**Fig. 2B**). Importantly, the level of apoptosis was slightly, but reproducibly, elevated in Rb/p130 DKO relative to Rb KO cultures (**Fig. 2B**, see below).

To determine whether combined mutations in Rb and p130 led to further disruption of the mitochondrial network seen in Rb deficient myotubes [36], we used MitoTracker®, a live-cell probe that accumulates in mitochondria with intact membrane potential. In DM-2 control myotubes, MitoTracker® staining revealed a uniform, net-like organization of mitochondria throughout the cytoplasm (**Fig. 2C**, top). However, both mgRb:Rb^−/−^ and mgRb:Rb^−/−^:p130^−/−^ myotubes exhibited relatively sparse cytosolic staining and strong perinuclear MitoTracker®-positive clusters in ∼70% of myotubes, indicative of autophagy (**Fig. 2C**). Importantly, there was no detectable difference in the level of perinuclear aggregation in single and DKO myoblasts. Together, these results indicate that loss of p130 does not ameliorate the Rb myogenic defect, but rather, exacerbates it by reducing myoblast survival prior to fusion.

### Efficient rescue of Rb/p130 DKO myoblast degeneration by Bcl-2, autophagy inhibitor and hypoxia

**Figure 3:**
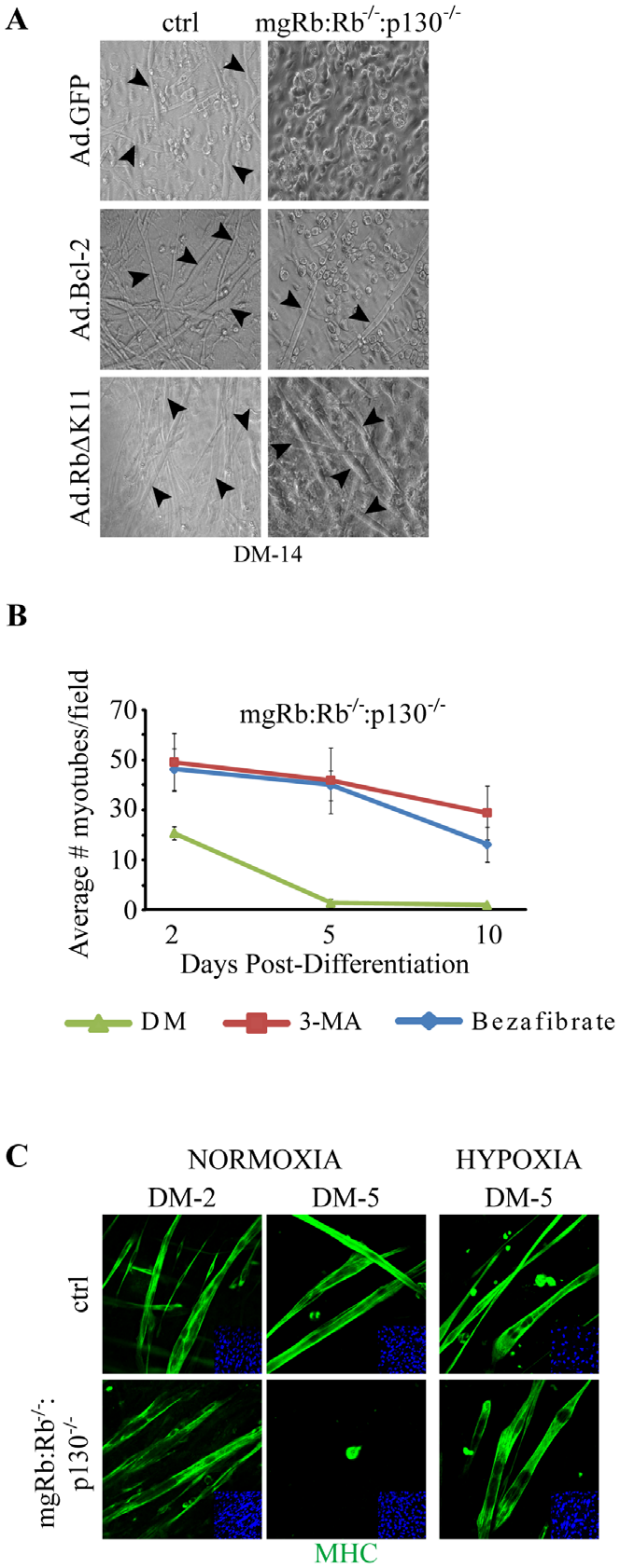
Rescue of mgRb:Rb^−/−^:p130^−/−^ myogenic defect by autophagy inhibitors and hypoxia. (A) Brightfield images of mgRb:Rb^−/−^:p130^−/−^ myoblasts transduced with Ad.GFP, Ad.Bcl-2 or Ad.RbΔK11 and then induced to differentiate for 14 days. Arrowheads point to myotubes. (B) Average number of mgRb:Rb^−/−^:p130^−/−^ myotubes following treatment with 3-MA, bezafibrate or DM as indicated. Counts are average ± s.d. of 6 fields at 200X (n = 3). (C) Immunostaining for MHC (green) in ctrl and mgRb:Rb^−/−^:p130^−/−^ cultures differentiated under normoxia or hypoxia. Note myotubes in mgRb:Rb^−/−^:p130^−/−^ cultures at DM-5 under hypoxia but not normoxia. Nuclei were counterstained with DAPI.

We next asked whether inhibition of autophagy would rescue muscle degeneration in mgRb:Rb^−/−^:p130^−/−^ DKO cultures as it does for Rb-deficient myotubes [36]. Remarkably, adenovirus mediated transduction of Bcl-2, which inhibits both apoptosis and autophagy [53], effectively rescued myogenic degeneration of mgRb:Rb^−/−^:p130^−/−^ DKO myotubes, leading to long twitching myotubes (**Fig. 3A**). This striking result demonstrates that when provided with a survival signal that counteracts the pro-apoptotic effect of Rb loss, myotube formation and maintenance does not require active participation of pRb and p130. We previously demonstrated that inhibition of autophagy by 3-methyladenine (3-MA), an antagonist of a class III phosphatidylinositol 3-kinase, Vps34, which is necessary for autophagic vesicle nucleation [54], also rescued the degeneration of Rb-deficient myotubes [36]. A single dose of 3-MA administered just prior to induction of differentiation prevented degeneration of mgRb:Rb^−/−^:p130^−/−^ DKO myotubes (**Fig. 3B**). The 3-MA-rescued myotubes twitched for weeks in culture and were indistinguishable from control myotubes (**Videos S1** and **S2**). The pan-PPAR agonist bezafibrate [55], [56], which induces mitochondrial biogenesis, also rescued the collapse of mgRb:Rb^−/−^:p130^−/−^ DKO myotubes with similar efficiency as 3-MA (**Fig. 3B**). As control, ectopic expression of a constitutively active, phospho-mutant pRb (Ad.Rb^ΔK11^) [57] prevented degeneration of mgRb:Rb^−/−^:p130^−/−^ DKO myotubes (**Fig. 3A**, bottom panel).

In addition to autophagy antagonists, hypoxia (∼1% O_2_) rescued the myogenic defect induced by loss of Rb by redirecting metabolism from oxidative phosphorylation to glycolysis [36]. To test whether hypoxia could also rescue the myogenic defect in Rb/p130 DKO myoblasts, mgRb:Rb^−/−^:p130^−/−^ and control cultures were induced to differentiate under hypoxia. Remarkably, despite the enhanced cell death and reduced myotube formation in mgRb:Rb^−/−^:p130^−/−^ cultures relative to mgRb:Rb^−/−^, hypoxia efficiently maintained the survival of DKO myotubes, leading to long, twitching myotubes (**Fig. 3C**). These results indicate that combined loss of Rb plus p130 reduces myoblast survival and myotube formation relative to Rb loss alone. Yet, when cell death is inhibited, DKO myotubes can differentiate as efficiently as Rb mutant or control cultures, demonstrating that neither factor is required for stimulating or maintaining the differentiation program.

### Inactivation of Rb but not p107 plus p130 leads to ectopic DNA synthesis and degeneration of primary myotubes

**Figure 4:**
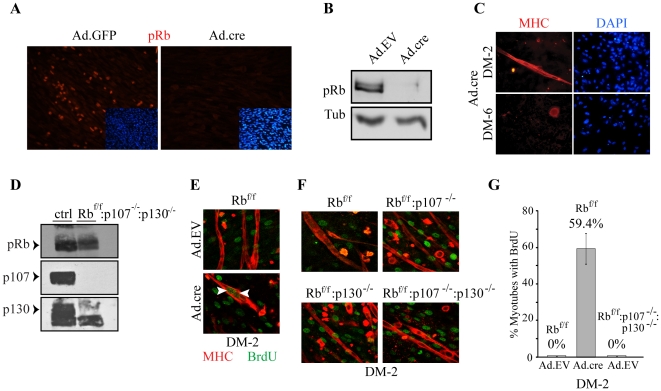
BrdU incorporation analysis of Rb^Δf^ versus p107^−/−^:p130^−/−^ myotubes. (A) Rb^f/f^ myoblasts were transduced with Ad.GFP or Ad.cre and 48 hr later were immunostained for pRb (red). Nuclei were counterstained with DAPI. (B) Rb^f/f^ myoblasts were transduced with Ad.EV or Ad.cre and immunoblotted for pRb 48 hr later. Tubulin served as loading control. (C) Rb^f/f^ myoblasts were transduced with Ad.cre, induced to differentiate for 2 or 6 days and immunostained for MHC (red). (D) Western blot analysis of pRb, p107 and p130 in skeletal muscle of E16.5 Rb^f/f^:p107^+/−^:p130^+/−^ (ctrl) and Rb^f/f^:p107^−/−^:p130^−/−^ fetuses. (E) Immunostaining for BrdU and MHC in Ad.EV and Ad.cre transduced Rb^f/f^ myoblasts at DM-2. Myoblasts were differentiated for 1 day, then exposed to 20 µM BrdU for an additional 16 hr in the presence of GM and stained for MHC (red) and BrdU (green). Arrowheads label BrdU positive nuclei within myotubes. (F) Immunostaining for BrdU and MHC in Rb^f/f^, Rb^f/f^:p107^−/−^, Rb^f/f^:p130^−/−^ and Rb^f/f^:p107^−/−^:p130^−/−^ myoblasts at DM-2. Myoblasts were differentiated for 1 day, then exposed to 20 µM BrdU for an additional 16 hr in the presence of GM and stained for MHC (red) and BrdU (green). Note absence of BrdU-positive nuclei in myotubes. (G) Quantification of BrdU incorporation in Ad.EV or Ad.cre transduced Rb^f/f^ and Rb^f/f^:p107^−/−^:p130^−/−^ myotubes at DM-2.

To characterize the functions of p107 and p130 during skeletal myogenesis in combination with Rb deletion, we isolated primary myoblasts from E16.5 Rb^f/f^:p107^−/−^:p130^−/−^ composite mutant embryos. In these mice, Rb exon 19 is flanked by loxP sites, allowing acute inactivation through Cre mediated excision [58]. Transduction of Rb^f/f^ myoblasts with Ad.Cre at a high multiplicity of infection (1300) led to loss of pRb expression in all cells as determined by immunostaining and immunoblotting (**Fig. 4A–B**), and to myotube degeneration (**Fig. 4C**). Deletion of p107 and p130 was detected by PCR (not shown) and confirmed by immunoblotting (**Fig. 4D**).

**Figure 5:**
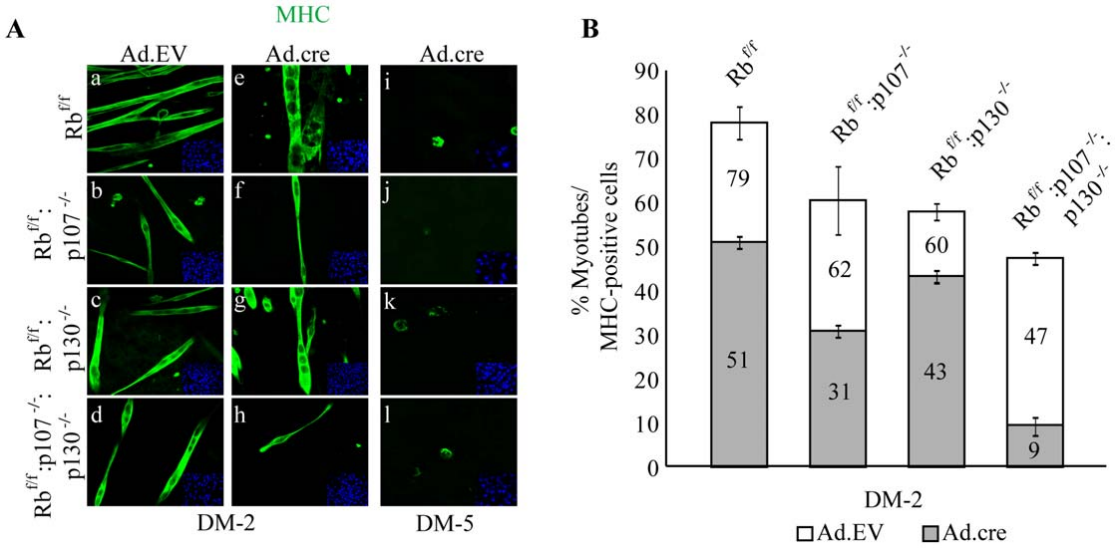
Differentiation potential of double and triple KO myoblasts. (A) Immunostaining for MHC (green) in Ad.EV and Ad.cre transduced Rb^f/f^, Rb^f/f^:p107^−/−^, Rb^f/f^:p130^−/−^ and Rb^f/f^:p107^−/−^:p130^−/−^ myoblast cultures at DM-2. Nuclei were counterstained with DAPI. (B) Quantification of percent multinucleated myotubes relative to total number of MHC-positive cells (myocytes plus myotubes) in Ad.EV and Ad.cre transduced Rb^f/f^, Rb^f/f^:p107^−/−^, Rb^f/f^:p130^−/−^ and Rb^f/f^:p107^−/−^:p130^−/−^ myoblasts at DM-2 under normoxia. Numbers within bars indicate % for the respective samples.

We first tested whether combined mutations in p107 and p130 resulted in deregulated cell proliferation in response to a differentiation signal. Interestingly, RNAi inhibition of Rb family members in C2C12 myotubes suggests that knock-down of p107 plus p130 does not cause cell cycle re-entry [59]. This could be due to incomplete knockdown of p107 and/or p130 by RNAi. In addition, C2C12 cells lack Arf [60], and therefore the observed effect could be confounded by deregulation of the ARF/MDM2/p53 pathway. To address the consequences of complete KO of p107 plus p130 in cells with an intact ARF-p53 pathway, we induced primary Rb^f/f^:p107^−/−^:p130^−/−^ and control myoblasts to differentiate and then re-stimulated with growth medium in the presence of BrdU. Rb^Δf^ mutant myotubes incorporated BrdU (**Fig. 4E, G**). In contrast, Rb^f/f^:p107^−/−^:p130^−/−^ double KO myotubes (which retain the two Rb^f/f^ alleles) did not (**Fig. 4F, G**). Thus, Rb loss prevents cell cycle exit during terminal differentiation and this function is unique to Rb. Notably, although Rb^f/f^:p107^−/−^:p130^−/−^ double mutant myotubes survived and twitched like control Rb-proficient cultures, we consistently observed reduced myotube formation in p107/p130 DKO cultures relative to control (e.g. **Fig. 5B**).

### Acute inactivation of Rb plus p107 or p130 leads to reduced myotube formation whereas TKO myoblasts form short bi-nuclear myocytes

**Figure 6:**
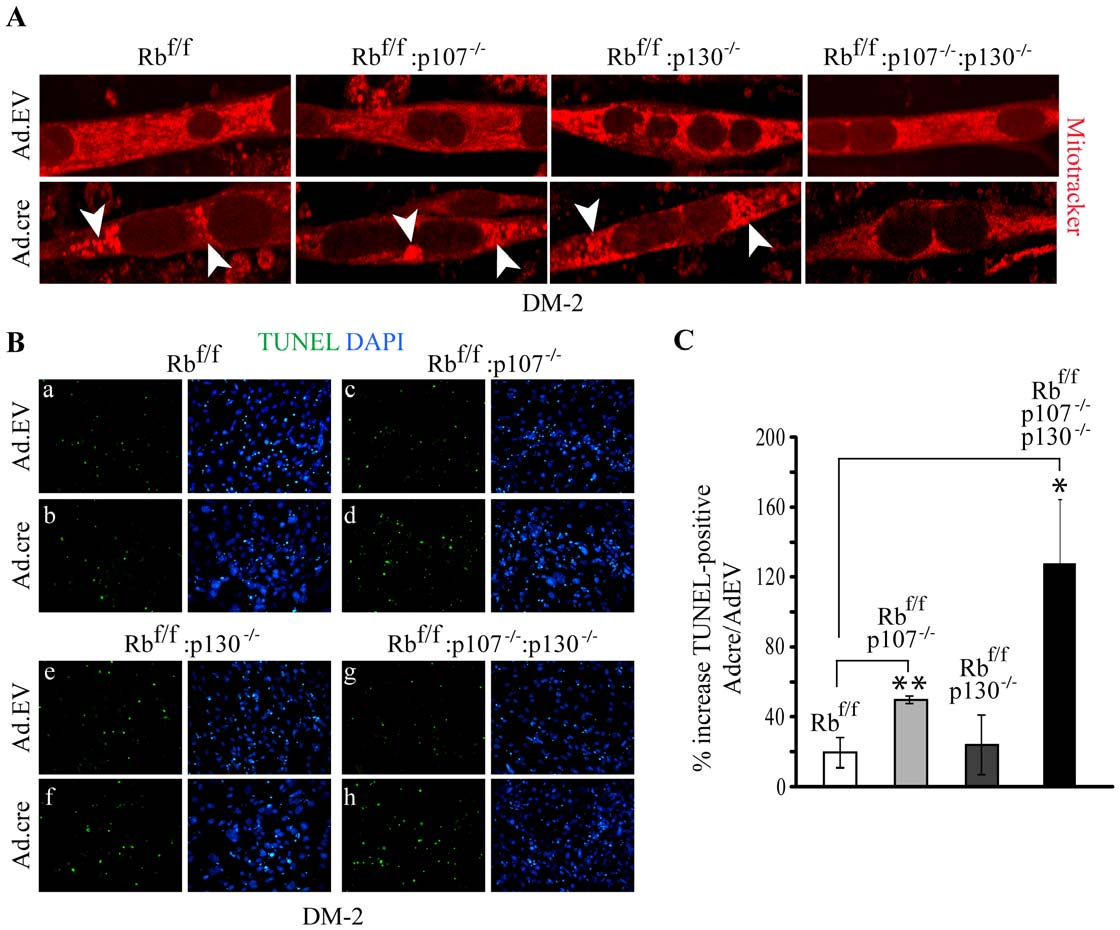
Increased apoptosis associated with differentiation of double and triple KO myoblasts. (A) Mitotracker® staining of Ad.EV and Ad.cre transduced Rb^f/f^, Rb^f/f^:p107^−/−^, Rb^f/f^:p130^−/−^ and Rb^f/f^:p107^−/−^:p130^−/−^ myoblasts at DM-2. Arrowheads point to large perinuclear aggregates in Ad.cre transduced myotubes. (B) TUNEL staining (green) of Ad.EV and Ad.cre transduced Rb^f/f^ (a–b), Rb^f/f^:p107^−/−^ (c–d), Rb^f/f^:p130^−/−^ (e–f) and Rb^f/f^:p107^−/−^:p130^−/−^ (g–h) cultures at DM-2. Nuclei were counterstained with DAPI. Note that TUNEL positive nuclei are outside myotubes. (C) Percent increase in TUNEL-positive cells in Ad.cre relative to Ad.EV transduced cultures. Error bars represent s.d. *-p<0.05 and **-p<0.07 t-test comparisons relative to Rb^f/f^.

To investigate the effect of Rb plus p107 and/or p130 on myogenesis, proliferating Rb^f/f^, Rb^f/f^:p130^−/−^, Rb^f/f^:p107^−/−^ and Rb^f/f^:p130^−/−^:p107^−/−^ myoblasts were transduced with Ad.cre or control empty vector (Ad.EV) and induced to differentiate 48 hr later (permitting pre-existing pRb protein degradation). Both Rb^Δf^:p130^−/−^ and Rb^Δf^:p107^−/−^ myoblasts differentiated to form short myotubes by DM-2 with slightly less myotubes in the Rb/p107 than the Rb/p130 DKO cultures (**Fig. 5A**). In addition to multinucleated myotubes, Rb^Δf^:p107^−/−^, and to a lesser extent Rb^Δf^:p130^−/−^ and Rb^Δf^ myoblasts formed elongated MHC-positive myocytes containing a single nucleus. Quantification of multinucleated myotubes relative to total MHC-positive cells (i.e. myocytes plus myotubes) revealed that Rb^Δf^, Rb^Δf^:p107^−/−^ and Rb^Δf^:p130^−/−^ cultures contained 51%, 31% and 43% myotubes relative to total MHC-positive cells, respectively (**Fig. 5B**). Rb^Δf^:p130^−/−^:p107^−/−^ TKO myoblasts formed primarily elongated myocytes that typically contained one or two nuclei (**Fig. 5A**, h; **Fig. 6A**, bottom right); some rare myotubes containing three nuclei were also observed (**Fig. S1**). The percentage of short bi-nuclear myotubes relative to total MHC-positive cells in the TKO cultures (single-nucleus myocytes plus binuclear myocytes or myotubes) was ∼9% (**Fig. 5B**).

### Reduced myotube formation in DKO and TKO cultures correlates with increased apoptosis in differentiating myoblasts

The reduced number of myotubes in Rb/p107, Rb/p130 and Rb/p107/p130 mutant cultures relative to Rb KO and control cultures could be due to intrinsic defects in differentiation or to excessive myoblast cell death, which would diminish the available pool of competent neighboring myocytes for fusion. To distinguish between these possibilities, we first assessed the level of mitochondrial perinuclear aggregation in differentiating myotubes. Following acute inactivation of Rb, transiently formed Rb^Δf^ myotubes, like mgRb:Rb^−/−^ myotubes, exhibited abnormal perinuclear aggregation of mitochondria (**Fig. 6A**, bottom left panel). The level of perinuclear mitochondrial aggregation in Rb/p107 and Rb/p130 DKO myotubes was similar to that observed in Rb KO cultures. In contrast, the short bi-nuclear TKO myocytes did not exhibit mitochondrial aggregation in the perinuclear region (**Fig. 6A**, bottom right panel).

TUNEL analysis revealed that differentiating TKO myoblasts underwent the highest level of apoptosis (119%) relative to the level of apoptosis in wild-type, followed by Rb/p107 (49%), Rb/p130 (24%) and Rb (19%) KO myoblasts (**Fig. 6B–C**). Thus, the reduced myotube formation (and increase in elongated myocytes) was directly proportional to the level of apoptosis in the various mutant cultures. Interestingly, it was reported that myoblasts seeded at low-density do not fuse under differentiation conditions but instead form elongated myocytes that undergo differentiation in the absence of fusion [60]. Thus, reduced myotube formation in the DKO and TKO cultures likely reflect, at least in part, the increased apoptosis and reduced number of competent myocytes available for fusion.

### Hypoxia efficiently rescues myotubes formed in the absence of Rb and p130 or p107, but not in the absence of all three Rb family proteins

**Figure 7:**
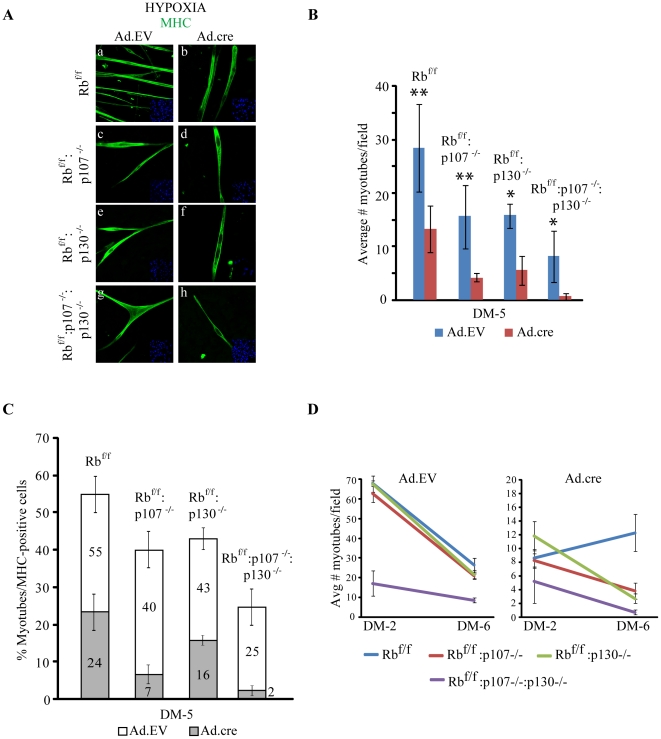
Differentiation of double and triple KO myoblasts under hypoxia. (A) Immunostaining for MHC (green) of Ad.EV (a,c,e,g) or Ad.cre (b,d,f,h) transduced Rb^f/f^, Rb^f/f^:p107^−/−^, Rb^f/f^:p130^−/−^ and Rb^f/f^:p107^−/−^:p130^−/−^ cultures at DM-5 in hypoxia. Inlets, DAPI staining for nuclei. (B) Quantification of myotube formation in Rb^f/f^, Rb^f/f^:p107^−/−^, Rb^f/f^:p130^−/−^ and Rb^f/f^:p107^−/−^:p130^−/−^ cultures transduced with Ad.EV or Ad.cre and induced to differentiate 48 hr later for 5 days. Counts represent the average number of myotubes at DM-5 of 6 representative fields (n = 4); error bars represent s.d. *-p<0.05 and **-p<0.07. (C) Quantification of percent multinucleated myotubes relative to total number of MHC-positive cells in Ad.EV or Ad.cre transduced Rb^f/f^, Rb^f/f^:p107^−/−^, Rb^f/f^:p130^−/−^ and Rb^f/f^:p107^−/−^:p130^−/−^ cultures at DM-5 under hypoxia. Numbers within bars indicate % for respective samples. (D) Quantification of myotube formation in Rb^f/f^, Rb^f/f^:p107^−/−^, Rb^f/f^:p130^−/−^ and Rb^f/f^:p107^−/−^:p130^−/−^ cultures transduced with Ad.EV or Ad.cre and induced to differentiate in hypoxia. Counts were conducted at DM-2 and DM-6. Error bars represent s.d.

**Figure 8:**
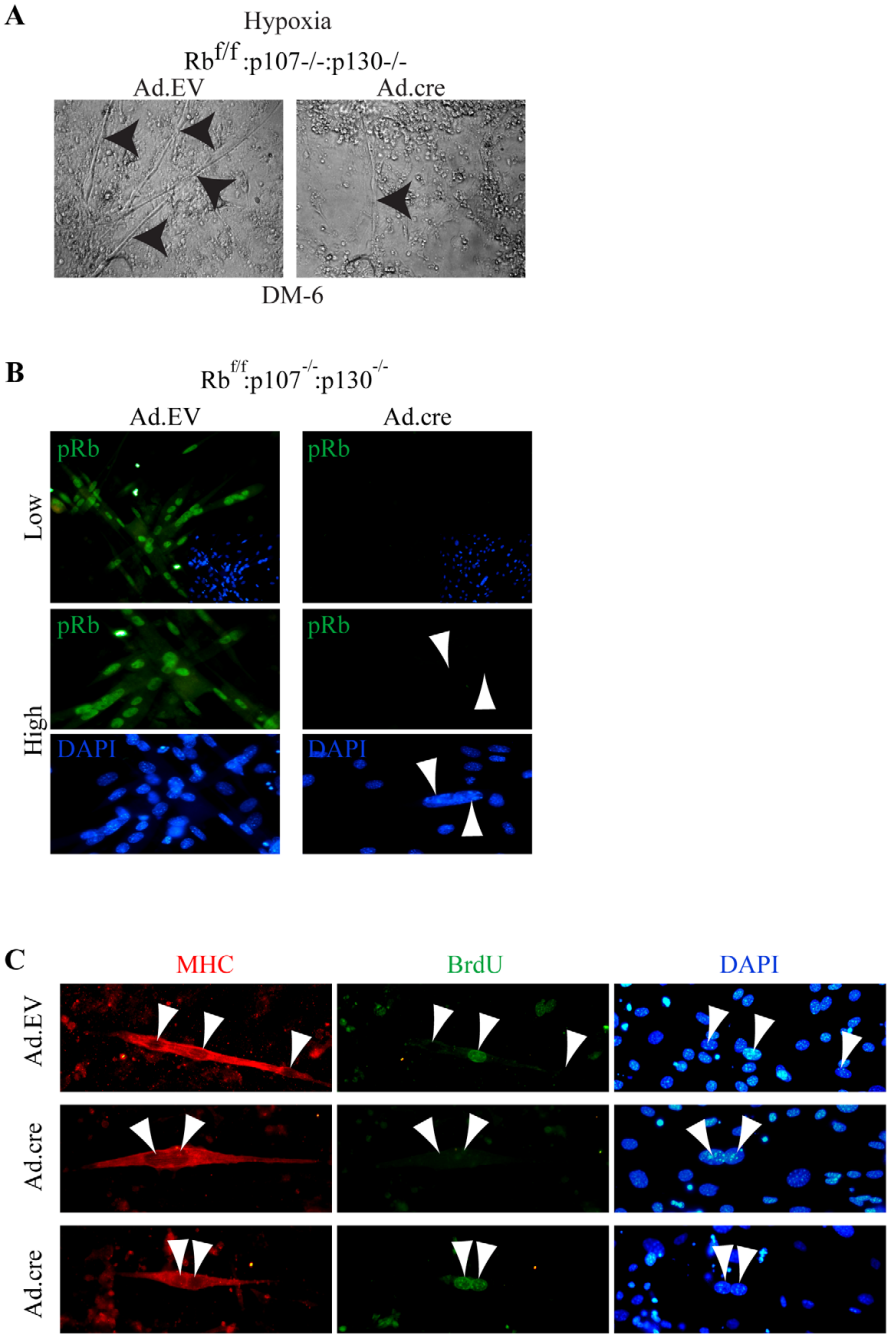
Evidence that bi-nuclear TKO myotubes originate from nuclear duplication, not cell fusion. (A) Bright-field images of Ad.EV and Ad.cre transduced Rb^f/f^:p107^−/−^:p130^−/−^ cultures at DM-6 in hypoxia. Arrowheads point to myotubes. Note the presence of a thin myotube in Ad.cre transduced culture. (B) Top row, low magnification image of Ad.EV or Ad.cre transduced Rb^f/f^:p107^−/−^:p130^−/−^ cultures immunostained for pRb (green) at DM-2 (400x). Bottom row, high magnification (630x) of Ad.EV or Ad.cre transduced Rb^f/f^:p107^−/−^:p130^−/−^ cultures induced to differentiate and then immunostained for pRb (green), demonstrating absence of detectable pRb within binuclear myocyte. Nuclei were counterstained with DAPI. Arrowheads point to nuclei in the myocyte. (C) Immunostaining for MHC (red) and BrdU (green) at DM-2 of Ad.EV and Ad.cre transduced Rb^f/f^:p107^−/−^:p130^−/−^ cultures, which were induced differentiate after equal mixing of BrdU^+^ labeled and BrdU^−^ myoblast populations. Note mixed BrdU^+^ and BrdU^−^ nuclei in control myotube (top panel) but only BrdU^−^:BrdU^−^ or BrdU^+^:BrdU^+^ nuclei in TKO myotubes (middle and bottom, respectively).

Hypoxia most effectively rescues the myogenic defect following acute inactivation of Rb [36]. To test whether hypoxia could prevent myotube degeneration following acute inactivation of Rb plus its relatives, Rb^f/f^, Rb^f/f^:p130^−/−^, Rb^f/f^:p107^−/−^ and Rb^f/f^:p130^−/−^:p107^−/−^ myoblasts were transduced with Ad.cre or Ad.EV and then either maintained under normoxia or shifted to hypoxic conditions. Under normoxia, no myotubes survived by DM-5 (**Fig. 5A**). However, under hypoxia, Rb^Δf^:p130^−/−^ and Rb^Δf^:p107^−/−^ myotubes survived and twitched, and appeared indistinguishable from Rb^Δf^ or control myotubes (**Fig. 7A–B**; **videos S3–5**). In striking contrast, elongated TKO myocytes/myotubes degenerated, forming ultra-thin myocytes/myotubes, very few of which nonetheless twitched (**video S6**). The ratio of myotubes to myocytes at DM-5 was similar, relative to DM-2 (compare **Fig. 5B** to **F** **ig. 7C**). To directly test this, we induced the various cultures to differentiate under hypoxia and then counted the number of myotubes at DM-2 and DM-6. As shown in **Figure 7D**, the ratio of myotubes at DM-2 and DM-6 was similar in the two DKO cultures indicating that once formed, myotubes can survive in hypoxia independently of pRb-p107 or pRb-p130 protein family. In contrast, very few TKO binuclear myocytes/myotubes survived at DM-6 under hypoxia, and they were ultra-thin and clearly abnormal (**Fig. 8A**). Thus, at least one pRb protein family is required for robust differentiation even under hypoxic conditions, which rescue Rb-deficient myotube degeneration.

### Short TKO binuclear myocytes originate primarily from acytokinetic mitosis and not fusion

If the bi-nuclear TKO myocytes originate from cell fusion, this would suggest that the entire Rb family is dispensable for this process. To address this possibility, we first asked whether the bi-nuclear TKO myocytes represented rare Rb^f/f^:p130^−/−^:p107^−/−^ myoblasts in which the Rb^f/f^ allele had not been deleted. However, upon staining, none of over 80 short binuclear myocytes expressed pRb (n = 2) (**Fig. 8B**). Next, we asked whether the elongated binuclear myocytes originated from cell fusion of TKO myocytes or from acytokinetic mitosis (i.e. absence of cytokinesis at the end of mitosis), which occurs normally in cardiac myocytes and other tissues [61]. To distinguish between these possibilities, proliferating TKO or control myoblasts (two independent cultures for each) were labeled with BrdU for 24 hr. The labeled cells were then mixed with an equal number of unlabeled TKO or control myoblasts, respectively, and induced to differentiate. Control cultures contained three populations of myotubes (BrdU^+^/BrdU^+^; BrdU^+^/BrdU^−^; BrdU^−^/BrdU^−^), with an average of 35% (of >200 myotubes) containing a mixture of BrdU^+^ and BrdU^−^ nuclei, indicative of cell fusion (n = 4) (**Fig. 8C**). In striking contrast, with the exception of one elongated bi-nuclear TKO myocyte with BrdU^+^/BrdU^−^ nuclei, more than 150 other bi-nuclear TKO myocytes contained either BrdU^−^/BrdU^−^ or BrdU^+^/BrdU^+^ nuclei, strongly suggesting that binuclear TKO myocytes originate primarily from acytokinetic mitosis, not from cell fusion. This is consistent with our observation that bi-nuclear TKO myocytes do not exhibit perinuclear aggregation of mitochondria as observed in Rb KO and DKO myotubes (**Fig. 6A**). Thus, these results suggest that efficient myocyte fusion requires the presence of at least one member of the Rb protein family.

## Discussion

We report that mutant myoblasts lacking Rb and one of its relatives, p107 or p130, can undergo robust myogenic differentiation under conditions we previously established whereby the survival defect in Rb-deficient myotubes is rescued by autophagy antagonists or hypoxia. In contrast, under the same conditions, combined mutations in all Rb protein family members, pRb, p107 and p130, severely abrogate myogenic differentiation. Thus, myoblast fusion and myotube survival require at least one Rb family member. We discuss our findings in the context of recent published work by another group demonstrating that various tissue-types can differentiate in the absence of Rb family and propose a tissue specific active/default model for Rb during cell fate determination and differentiation.

It is commonly thought that the tumor suppressor pRb plays at least two independent functions during differentiation: control of cell division and apoptosis through inhibition of E2F responding genes, and stimulation of the differentiation program through activation of differentiation factors. However, we recently showed that survival factors, autophagy inhibitors and hypoxia can prevent the degeneration of Rb-deficient muscles, leading to contracting myotubes [36]. While these observations challenge the notion that pRb is actively required to stimulate the differentiation program, the possibility that p107 and/or p130 partially compensate for pRb during differentiation was not ruled out. Specifically, p107 or p130 could account for the ability of Rb-deficient myoblasts to fuse to form short myotubes prior to degeneration. In addition, it was unresolved whether p130 compensates or counteracts pRb during differentiation [48]. Here, we addressed these issues by analyzing primary myoblasts with composite mutations in the Rb gene family. We showed that combined mutations in pRb plus p107 or pRb plus p130 increased apoptosis in myoblasts, and accordingly, reduced the number of DKO myotubes. p107 was clearly more critical than p130 in preventing apoptosis of Rb-deficient myoblasts during differentiation. Nevertheless, the myotubes that formed in the absence of Rb and either p107 or p130 survived and twitched under hypoxia or following treatment with autophagy-antagonists.

Several studies suggest that the Rb family may affect differentiation and lineage commitment by transcriptionally regulating the expression of differentiation factors such as PPARγ and PGC-1α, by sequestering inhibitors of differentiation like ID2, HDAC, EID1 and RBP2 [6], [7], [8], [9], [10], [11], or by binding and stimulating differentiation factors such as CBFA1 during osteoblast differentiation, C/EBPβ during adipocyte differentiation and MyoD and myogenin during myogenesis [12], [13], [14], [15], [16], [17]. A recent genome-wide mammalian protein-protein interaction analysis independently demonstrated that human RB1 and p130 but not p107 interact with MYOD1, RUNX2 and C/EBP [62]. p107 does not seem to interact with MyoD or any other differentiation factor, which is consistent with its reduced expression during differentiation. Thus, the ability of Rb/p130 DKO myoblasts to fully differentiate when treated with autophagy inhibitors or hypoxia is remarkable and questions the notion that the Rb family is actively required for differentiation. Possibly, interaction of pRb and p130 with MyoD may be required for cell survival or cell cycle exit, but it does not seem essential for differentiation *per se*.

While we observed a quantitative reduction in myotube formation between single KO and double KO myoblasts, there was a qualitative difference between DKO and TKO cultures. TKO myoblast cultures exhibited excessive myoblast death and primarily formed elongated, bi-nuclear myocytes and some rare *bona fide* myotubes. Using nuclear labeling and mixing experiments, we present evidence suggesting that the bi-nuclear myocytes originate from acytokinetic mitosis, not cell fusion. Under hypoxia, the short TKO myocytes/myotubes became abnormally thin, yet some rare myocytes/myotubes persisted and twitched. The appearance of rare twitching TKO myotubes with three nuclei suggests that some cell fusion can occur, albeit inefficiently, in the absence of all three Rb protein family. We conclude that the presence of a single member of the Rb protein family is required for efficient myocyte fusion, survival and differentiation even under hypoxia. However, we cannot rule out the possibility that under certain conditions, yet to be identified, TKO myoblasts might fuse to form normal-like twitching myotubes.

After submission of this manuscript, the Sage group reported that Rb-family TKO embryos form various tissues containing multiple cell lineages. However, skeletal myotubes were completely absent in cross sections through back/axial muscles of TKO embryos [63]. Although more detailed analyses of the TKO muscle defect is needed, these observations are consistent with our *in vitro* results demonstrating an autonomous requirement for the Rb family for myogenesis, even under hypoxia.

We propose the following active/default model for pRb. In this model, pRb is actively required for differentiation of certain tissues, whereas other tissues, including adipose and those that develop early in embryogenesis before Rb family gene expression is observed [24], can differentiate in the absence of Rb, i.e. as a default pathway. Indeed, it was recently shown that Rb status dictates fate choice between osteogenic and adipogenic differentiation by positively regulating the osteogenic factor RUNX2 and negatively regulating the adipogenic factor PPARγ [6]. Likewise, Rb (and p107) is required for differentiation of adipocytes to white adipose tissue by suppressing PGC-1α expression, whereas the default differentiation in the absence of Rb or p107 is brown fat [46]. In TKO hematopoietic stem cells, myeloid progenitors hyper-proliferate whereas lymphoid progenitors are ablated [64]. While Rb may be required solely for survival of muscle, bone, white fat and lymphoid cells, these studies suggest that it is actively engaged in sequestering inhibitors of differentiation or stimulating expression or activity of differentiation factors in stem/progenitors cells at the stage of bifurcation into different cell lineages. Whether Rb dictates cell fate choice during differentiation of multipotent stem cells in the somite or in Pax3^+^/Pax7^+^ muscle stem cells in the dermomyotome [65] is yet to be determined. Such instructive functions by the Rb family during cell fate determination may no longer be required once a cell becomes committed to a specific lineage, and the major function of Rb in committed cells might be to allow proper cell cycle exit and survival; a function that can be bypassed by survival factors or hypoxia. The diminished ability of TKO myoblasts to differentiate and survive may be a consequence of complete deregulation of the E2F protein family, which may create conditions that are incompatible with differentiation, or reflect a requirement for Rb protein family in myoblast fusion and myotube survival, which cannot be rescued by hypoxia.

Finally, Rb but not p107 or p130 is often lost in cancer. As a potential basis for this observation, it was suggested that Rb inactivation is uniquely required for cancer progression because only after its loss can tumor cells escape senescence under oncogenic stress [39]. Conversely, in response to p16^ink4a^ over-expression, mutations in p107 plus p130 allow cells to escape cell cycle inhibition as efficiently as mutations in Rb [38], suggesting that these factors have similar functions in cell cycle exit in response to CDK4/6 inhibition. Here, using KO myoblasts, we demonstrated that Rb, but not p107 plus p130, is uniquely required for cell cycle exit during terminal differentiation of primary myoblasts. Thus, the exclusive role of Rb but not its relatives in certain cancers may be due to its unique role in enforcing cell cycle exit during terminal differentiation. Likely, disruption of either function of pRb, senescence or cell cycle exit during differentiation, can lead to neoplastic transformation depending on the cellular and oncogenic context.

## Methods

### Mouse maintenance, genotyping & timed-pregnancy

Experiments were performed in accordance with guidelines of the Canadian Council on Animal Care and approved by the TGRI-UHN Animal Care Committee, Ontario (Approval ID: AUP1050). Mice were genotyped using DNA extracted from tail biopsies and the following primers: mgRb:Rblox – forward 5′-ATTTCAGAAGGTCTGCCAAC, reverse 5′-AGAGCAGGCCAAAAGCCAGGA; Rb mutant, AATTGCGGCCGCATCTGCATCTTTATCGC and GAAGAACGAGATCAGCAG; Rb wild-type AATTGCGGCCGCATCTGCATCTTTATCGC and CCCATGTTCGGTCCCTAG; Rb^floxed^ (Rb18 + Rb19E) GGCGTGTGCCATCAATG and CTCAAGAGCTCAGACTCATGG; p130 wild-type ACGGATGTCAGTGTCACG and TACATGGTTTCCTTCAGCGG; p130 mutant ACGGATGTCAGTGTCACG and GAAGAACGAGATCAGCAG; p107 wild-type TCGTGAGCGGATAGAAAG and GTGTCCAGCAGAAGTTA; p107 mutant TCGTGAGCGGATAGAAAG and CCGCTTCCATTGCTCAGCGG. For timed-pregnancies, mice were mated overnight and the day of vaginal plug observation was considered E0.5.

### Isolation of myoblasts and cell culture

Skeletal muscles from limbs E16.5–E17.5 embryos obtained following timed-pregnancy were used to generate primary myoblast cultures. More than 700 embryos were used in this study. To maintain consistency between experiments, primary myoblasts were induced to differentiate at passage two. Muscle tissues were digested for 20 min at 37°C in 80 µl solution containing 1.5 U/ml collagenase IV (Sigma), 2.4 U/ml Dispase (Roche) and 5 mM CaCl_2_, gently triturated and plated onto 60 mm collagen-I coated culture dishes. Primary myoblasts were maintained in Growth Medium (GM) - HAM's-F10 (Lonza) supplemented with 20% FBS (PAA) and 2.5 ng/ml basic fibroblast growth factor (bFgf) (Sigma) - in a humidified incubator at 5% CO_2_ and 37°C. To induce differentiation, myoblasts were washed once in 1x Phosphate Buffered Saline (PBS) and shifted to Differentiation Medium (DM) - Dulbecco's modified Eagle's medium (DMEM, high-glucose and sodium pyruvate) (Sigma) supplemented with 3% Horse Serum (PAA) [66]. For drug treatment, a single dose of 3-methyladenine (5 mM) was added upon differentiation. Bezafibrate (500 µM) was refreshed every other day.

### BrdU DNA synthesis assay

Post-differentiation day 1 myotube cultures were re-stimulated in GM supplemented with 20 µM BrdU for 16 h before fixation with 3.7% formaldehyde (10 min). For BrdU-labeled cell mixing experiments, cultures were divided into two equal populations and transduced with Ad.cre or Ad.EV (see Ad.cre transduction methods). One of the two populations was fed 20 µM for 36 hr. BrdU was then removed, both populations trypsinized, mixed in equal proportions and plated in GM. Differentiation was induced 12–16 hr later. Cultures were permeabilized using 0.3% Triton X-100 for 10 min, treated with 2N HCl for 25 min, and neutralized with two washes of 0.5 M sodium borate, pH 8.5 for 5 min. After blocking in 1.0% BSA for 20 min, primary anti-MHC antibody; 1:50 (clone MY-32, Sigma) for 1 hr, cells were washed 3x, 3 min each with PBS. Secondary antibody: fluorescein-conjugated (Alexa Fluor 563 (Red) - Invitrogen). BrdU was detected using anti-BrdU antibody directly conjugated to FITC as per manufacturer's protocol (BD Biosciences). Images were captured using an Axioskop2 fluorescent microscope (Carl Zeiss Inc.).

### Immunofluorescence

500,000 primary myoblasts were seeded on 22 mm round Collagen-I coated coverslips (BD Biosciences) and induced to differentiate. Cells were fixed in 3.7% formaldehyde, permeabilized in 0.3% Triton X-100 and blocked for 20 min in 1% BSA/PBS at room temperature. Primary antibodies: MHC, 1:50 (clone MY-32, Sigma), pRb, 1:100 (BD Biosciences), were incubated on samples for 1 hr at room temperature. Secondary antibodies, fluorescein-conjugated (Alexa Fluor 563, Alexa Fluor 488 - Invitrogen) were added for 45 min. Nuclei were counterstained with DAPI (Invitrogen) for 10 min and mounted in fluorescent mounting media (Dako). Confocal images of 0.5 µm sections were captured at room temperature using a 40x or 63x c-apochromat objective lens (water)/1.2NA using a Zeiss LSM510 META confocal microscope (Carl Zeiss Inc.) and Zeiss AIM 3.2 acquisition software. Adobe Photoshop CS2 was used to overlay images.

### Western Blot Analysis

Cells were lysed on ice in K4IP buffer (50 mM HEPES, pH 7.5, 0.1% Tween-20, 1 mM EDTA, 2.5 mM EGTA, 150 mM NaCl, 1.0 mM DTT, 10% Glycerol) containing protease inhibitors (Sigma). Antibodies were used for 3 hr at room temperature or overnight at 4°C: α/β-tubulin, 1:4000 (Cell Signaling), pRb, 1:1000 (BD Biosciences), p130 (Santa Cruz), p107 (Santa Cruz). Secondary antibodies were HRP-linked anti-IgG, 1:2000 (Cell Signaling) for 1.5 hr in blocking buffer and HRP activity was detected using SuperSignal West Dura chemiluminescent substrate (Pierce) and captured by X-ray film. Films were digitized using a Canon scanner.

### MitoTracker® Red CMXRos and TUNEL Assays

Mitochondria membrane potential was detected using MitoTracker® Red CMXRos according to manufacturer's protocol (Molecular Probes). TUNEL analysis on section was performed as described [66]. For TUNEL analysis of tissue culture, differentiating myoblasts on collagen-I coated coverslips were fixed in 3.7% formaldehyde for 10 min, washed three times in PBS, permeabilized with 0.3% Triton X-100 solution and washed 3x in PBS. Subsequently, 30U Terminal Deoxynucleotidyl Transferase (TdT) (Fermentas) was added to 50 µl TUNEL-Label Solution (Roche). Nuclei were counterstained with DAPI (Invitrogen) for 10 min and mounted in fluorescent mounting media (Dako).

### Adenovirus-cre Transductions

Adenoviruses were amplified in 293T cells maintained in DMEM plus 10% FBS and Penicillin/Streptomycin (Sigma). For Adenovirus-cre (Ad.cre; Vector Biolabs) infection, Rb^f/f^ primary myoblasts were infected with multiplicity of infection (MOI) of 1300, which completely eliminated Rb expression. At lower MOI (<1000), some pRb positive nuclei within myoblasts were detected. For transductions, 50,000 cells (96-well) or 450,000 cells (22 mm round coverslip) were seeded and 8 hr later transduced with Ad.cre or Ad.EV in 50 µl or 600 µl GM, respectively. After 18 hr, medium was re-freshed for additional 24 hr. Cells were rinsed once using 1x PBS and switched to DM. The following adenovirus vectors were kindly provided by Marco Crescenzi - Ad.Bcl-2 - Dept. of Environment and Primary Prevention, Higher Institute of Health, Viale Regina Elena 299, 00161 Roma, Italy; Ad.EV - Genzyme Corporation, 31 New York Ave, P.O. Box 9322, Framingham, MA 01701-9322; David S. Park – Ad.Rb^ΔK11^ and Ad.p27 - Department of Cellular and Molecular Medicine, University of Ottawa, Ottawa, Ontario, Canada K1H 8M5.

### Histology and Immunofluorescence on embryo sections

Briefly, embryos were fixed in 4% paraformaldehyde at 4°C overnight, dehydrated, and embedded in paraffin. Sections (8 µm) were cut using a Reichert Ultracut E microtome. For histology, sections were stained with hematoxylin and eosin. For immunofluorescence staining, samples were de-paraffinized, hydrated and subjected to antigen retrieval by boiling in 10 mmol/L sodium citrate (pH 6.0) for 10 min in microwave followed by 30 min gradual cooling at room temperature. Slides were incubated with primary troponin T, 1:200 (clone c-18, Santa Cruz) antibody or MHC, 1:50 (clone MY-32, Sigma) in a humidified chamber at 4°C overnight. Secondary antibody, fluorescein-conjugated (Alexa Fluor 488 - Invitrogen) was added for 1 hr. Nuclei were counterstained with DAPI (Invitrogen) for 10 min and mounted in fluorescent mounting media (Dako). Images were captured using an Axioskop2 fluorescent microscope (Carl Zeiss Inc.). Confocal images of 0.5 µm sections were captured at room temperature using a 40x or 63x c-apochromat objective lens (water)/1.2NA using a Zeiss LSM510 META confocal microscope (Carl Zeiss Inc.) and Zeiss AIM 3.2 acquisition software. Adobe Photoshop CS2 was used to overlay images.

### Brightfield Images and Videos

Brightfield images and videos were captured at room temperature using 20x or 40x air-objective lenses on a Nikon TE200 microscope (Nikon) fitted with a Hamamatsu CCD digital camera. Images were acquired using SimplePCI imaging software (Hamamatsu). Adobe Photoshop CS2 was used to enhance clarity and contrast using same parameters for control and experimental samples.

## Supporting Information

Figure S1 **A rare TKO myotube containing 3 nuclei.** Immunostaining for pRb (green) of Ad.EV and Ad.cre transduced Rb^f/f^:p107^−/−^:p130^−/−^ cultures at DM-2. Top row, low exposure images demonstrating absence of detectable pRb in Ad.cre transduced culture. Bottom row, high exposure images to highlight a short myotube containing 3 nuclei, which is devoid of detectable nuclear pRb, in Ad.cre transduced Rb^f/f^:p107^−/−^:p130^−/−^ culture. Nuclei counterstained with DAPI.
(TIF)

Video S1 **Twitching wild-type myotubes treated with 3-MA.** Control wild-type myoblasts were induced to differentiate and treated with 3-MA and video captured at DM-5.
(SWF)

Video S2 **Twitching mgRb:Rb** ^−**/**−^ **:p130** ^−**/**−^ **myotubes treated with 3-MA.** mgRb:Rb^−/−^:p130^−/−^ myoblasts were induced to differentiate and treated with 3-MA and video captured at DM-5.
(SWF)

Video S3 **Twitching Ad.cre transduced Rb^Δf^ myotubes under hypoxia.** Rb^f/f^ myoblasts were transduced with Ad.cre, induced to differentiate after 48 hr, transferred to hypoxia and video captured at DM-5.
(SWF)

Video S4 **Twitching Ad.cre transduced Rb^Δf^:p107** ^−**/**−^ **myotubes under hypoxia.** Rb^f/f^:p107^−/−^ myoblasts were transduced with Ad.cre, induced to differentiate after 48 hr, transferred to hypoxia and video captured at DM-5.
(SWF)

Video S5 **Twitching Ad.cre transduced Rb^Δf^:p130−/− myotubes under hypoxia.** Rb^f/f^:p130^−/−^ myoblasts were transduced with Ad.cre, induced to differentiate after 48 hr, transferred to hypoxia and video captured at DM-5.
(SWF)

Video S6 **Twitching Ad.cre transduced Rb^Δf^:p107** ^−**/**−^ **:p130** ^−**/**−^ **myotube/myocyte under hypoxia.** Rb^f/f^:p107^−/−^:p130^−/−^ myoblasts were transduced with Ad.cre, induced to differentiate after 48 hr, transferred to hypoxia and video captured at DM-5.
(SWF)
