# Supplementary figures and images for: Critical Role of the Rb Family in Myoblast Survival and Fusion
Source: PLoS One. 2011 Mar 10;6(3):e17682. doi: 10.1371/journal.pone.0017682 (PMC3053373; doi:10.1371/journal.pone.0017682)

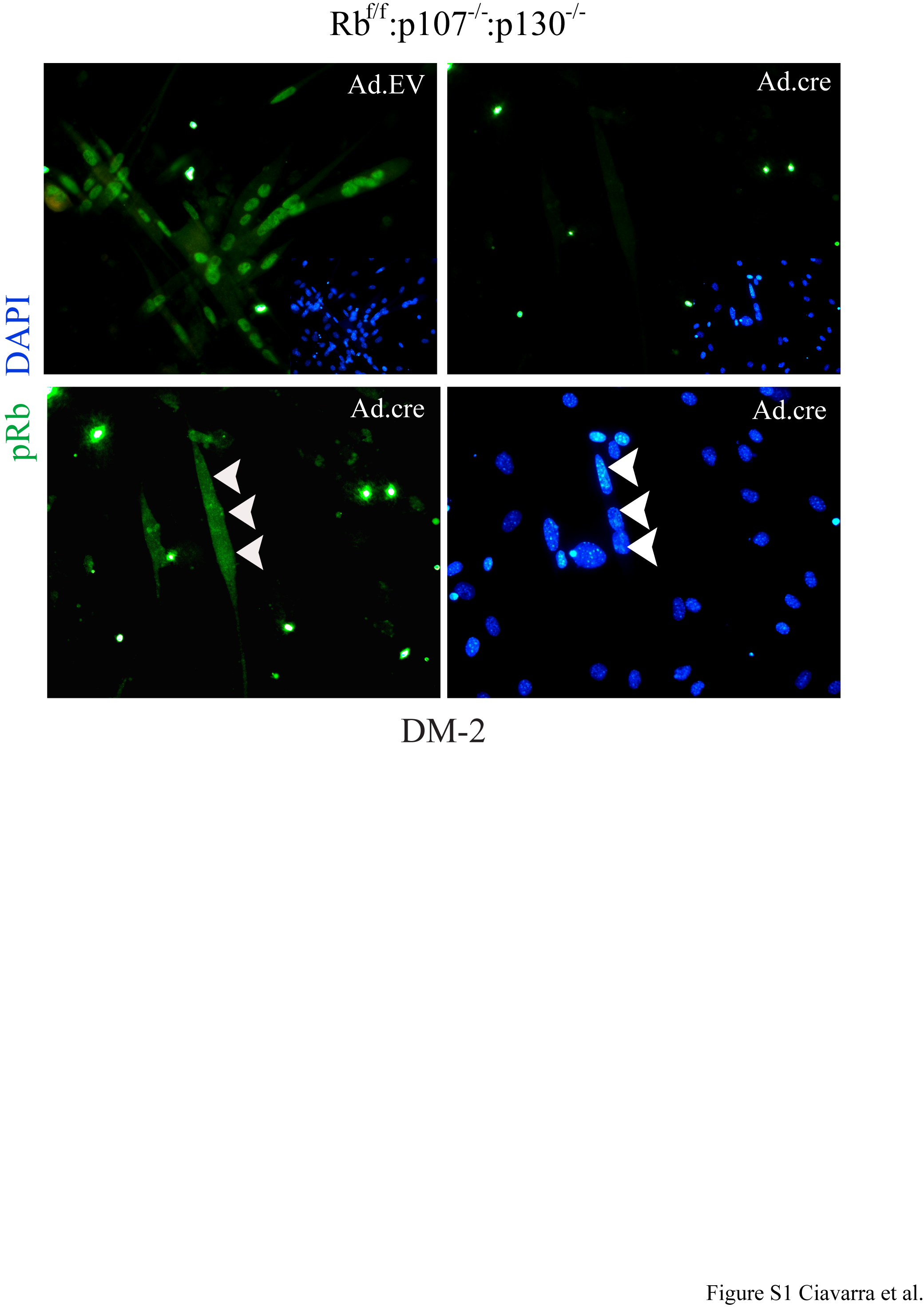

Supplement: Figure S1 — A rare TKO myotube containing 3 nuclei. Immunostaining for pRb (green) of Ad.EV and Ad.cre transduced Rbf/f:p107−/−:p130−/− cultures at DM-2. Top row, low exposure images demonstrating absence of detectable pRb in Ad.cre transduced culture. Bottom row, high exposure images to highlight a short myotube containing 3 nuclei, which is devoid of detectable nuclear pRb, in Ad.cre transduced Rbf/f:p107−/−:p130−/− culture. Nuclei counterstained with DAPI. (TIF) [file pone.0017682.s001.tif]
